# 3-(3-Fluoro­phenyl­sulfin­yl)-2,4,6-trimethyl-1-benzofuran

**DOI:** 10.1107/S1600536811043716

**Published:** 2011-10-29

**Authors:** Pil Ja Seo, Hong Dae Choi, Byeng Wha Son, Uk Lee

**Affiliations:** a Department of Chemistry, Dongeui University, San 24 Kaya-dong Busanjin-gu, Busan 614-714, Republic of Korea; b Department of Chemistry, Pukyong National University, 599-1 Daeyeon 3-dong, Nam-gu, Busan 608-737, Republic of Korea

## Abstract

In the title compound, C_17_H_15_FO_2_S, the 3-fluoro­phenyl ring makes a dihedral angle of 78.38 (4)° with the mean plane of the benzofuran fragment. In the crystal, mol­ecules are linked by weak C—H⋯O and C—H⋯π inter­actions. The crystal structure also exhibits a slipped π–π inter­action between the furan and benzene rings of neighbouring mol­ecules [centroid–centroid distances = 3.628 (2) Å, inter­planar distance = 3.417 (2) Å and slippage = 1.219 (2) Å].

## Related literature

For the pharmacological activity of benzofuran compounds, see: Aslam *et al.* (2009 ▶); Galal *et al.* (2009 ▶); Khan *et al.* (2005 ▶). For natural products with benzofuran rings, see: Akgul & Anil (2003 ▶); Soekamto *et al.* (2003 ▶). For the crystal structures of related compounds, see: Choi *et al.* (2010*a* ▶,*b* ▶). 
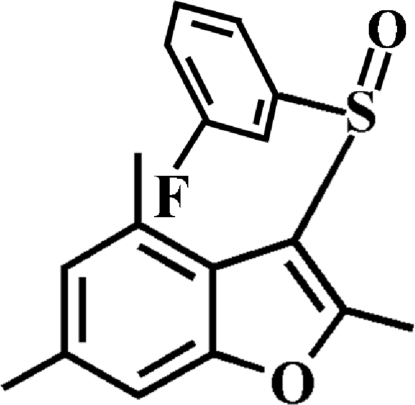


## Experimental

### 

#### Crystal data

C_17_H_15_FO_2_S
*M* *_r_* = 302.35
Triclinic, 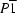

*a* = 6.8561 (2) Å
*b* = 8.0705 (2) Å
*c* = 13.9069 (3) Å
α = 103.719 (1)°
β = 91.280 (1)°
γ = 106.973 (1)°
*V* = 711.50 (3) Å^3^
*Z* = 2
Mo *K*α radiation
μ = 0.24 mm^−1^
*T* = 173 K
0.36 × 0.20 × 0.17 mm

#### Data collection

Bruker SMART APEXII CCD diffractometer
Absorption correction: multi-scan (*SADABS*; Bruker, 2009 ▶) *T*_min_ = 0.919, *T*_max_ = 0.960
12726 measured reflections
3257 independent reflections
2971 reflections with *I* > 2σ(*I*)
*R*_int_ = 0.023

#### Refinement

*R*[*F* ^2^ > 2σ(*F* ^2^)] = 0.035
*wR*(*F* ^2^) = 0.098
*S* = 1.06
3257 reflections
193 parameters
H-atom parameters constrained
Δρ_max_ = 0.31 e Å^−3^
Δρ_min_ = −0.29 e Å^−3^

### 

Data collection: *APEX2* (Bruker, 2009 ▶); cell refinement: *SAINT* (Bruker, 2009 ▶); data reduction: *SAINT*; program(s) used to solve structure: *SHELXS97* (Sheldrick, 2008 ▶); program(s) used to refine structure: *SHELXL97* (Sheldrick, 2008 ▶); molecular graphics: *ORTEP-3* (Farrugia, 1997 ▶) and *DIAMOND* (Brandenburg, 1998 ▶); software used to prepare material for publication: *SHELXL97*.

## Supplementary Material

Crystal structure: contains datablock(s) global, I. DOI: 10.1107/S1600536811043716/mw2033sup1.cif

Structure factors: contains datablock(s) I. DOI: 10.1107/S1600536811043716/mw2033Isup2.hkl

Supplementary material file. DOI: 10.1107/S1600536811043716/mw2033Isup3.cml

Additional supplementary materials:  crystallographic information; 3D view; checkCIF report

## Figures and Tables

**Table 1:** Hydrogen-bond geometry (Å, °) *Cg*2 is the centroid of the C2–C7 benzene ring.

*D*—H⋯*A*	*D*—H	H⋯*A*	*D*⋯*A*	*D*—H⋯*A*
C11—H11*B*⋯O2^i^	0.98	2.30	3.2727 (18)	172
C10—H10*A*⋯*Cg*2^ii^	0.98	2.74	3.655 (2)	155

Symmetry codes: (i) 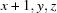; (ii) 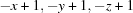.

**Fig. 1.**
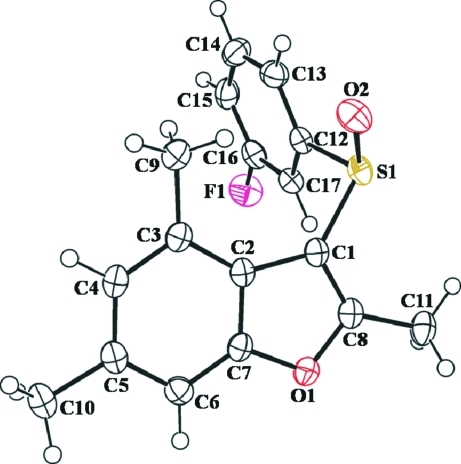
The molecular structure of the title compound with the atom numbering scheme. Displacement ellipsoids are drawn at the 50% probability level. H atoms are presented as small spheres of arbitrary radius.

**Fig. 2.**
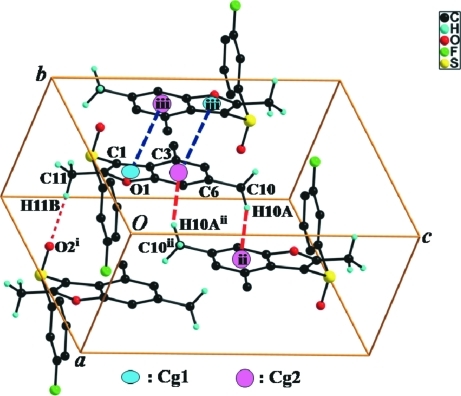
A view of the C–H···O, C–H···π and π–π interactions (dottedlines) in the crystal structure of the title compound. H atoms non-participating in hydrogen-bonding were omitted for clarity. [Symmetry codes: (i) x + 1 , y, z; (ii) - x + 1, - y + 1, - z + 1; (iii) -x + 1, - y + 2, - z + 1.]

## supplementary crystallographic information

### Comment

Substituted benzofuran derivatives have drawn much attention due to their valuable pharmacological properties such as antibacterial and antifungal, antitumor and antiviral, and antimicrobial activities (Aslam *et al.*, 2009, Galal *et al.*, 2009, Khan *et al.*, 2005). These benzofuran derivatives occur in a wide range of natural products (Akgul & Anil, 2003; Soekamto *et al.*, 2003). As a part of our ongoing study of benzofuran derivatives containing either 3-(4-fluorophenylsulfinyl) (Choi *et al.*, 2010*a*) or 3-(4-chlorophenylsufinyl) (Choi *et al.*, 2010*b*) substituents, we report herein the crystal structure of the title compound.

In the title molecule (Fig. 1), the benzofuran unit is essentially planar, with a mean deviation of 0.019 (1) ° from the least-squares plane defined by the nine constituent atoms. The dihedral angle between the 3-fluorophenyl ring and the mean plane of the benzofuran fragment is 78.38 (4)°. The crystal packing (Fig. 2) is stabilized by weak intermolecular C–H···O and C–H···π interactions (Table 1) as well as by a weak slipped π–π interaction between the furan and benzene rings of adjacent molecules with a Cg1···Cg2^iii^ distance of 3.628 (2) Å and an interplanar distance of 3.417 (2) Å resulting in a slippage of 1.219 (2) Å (Cg1 and Cg2 are the centroids of the C1/C2/C7/O1/C8 furan ring and the C2-C7 benzene ring, respectively).

### Experimental

77% 3-chloroperoxybenzoic acid (291 mg, 1.3 mmol) was added in small portions to a stirred solution of 3-(3-fluorophenylsulfanyl)-2,4,6-trimethyl 1-benzofuran (343 mg, 1.2 mmol) in dichloromethane (40 mL) at 273 K. After being stirred at room temperature for 4h, the mixture was washed with saturated sodium bicarbonate solution and the organic layer was separated, dried over magnesium sulfate, filtered and concentrated at reduced pressure. The residue was purified by column chromatography (hexane-ethyl acetate, 2:1 v/v) to afford the title compound as a colorless solid [yield 76%, m.p. 406-407 K; *R*_f_ = 0.55 (hexane-ethyl acetate, 4:1 v/v)]. Single crystals suitable for X-ray diffraction were prepared by slow evaporation of a solution of the title compound in benzene at room temperature.

### Refinement

All H atoms were positioned geometrically and included as riding contributions with C–H = 0.95 Å for aryl and 0.98 Å for methyl H atoms. *U*_iso_(H) =1.2*U*_eq_(C) for aryl and 1.5*U*_eq_(C) for methyl H atoms.

### Crystal data

**Table:**

C_17_H_15_FO_2_S	*Z* = 2
*M**_r_* = 302.35	*F*(000) = 316
Triclinic, *P*1	*D*_x_ = 1.411 Mg m^−^^3^
Hall symbol: -P 1	Mo *K*α radiation, λ = 0.71073 Å
*a* = 6.8561 (2) Å	Cell parameters from 7579 reflections
*b* = 8.0705 (2) Å	θ = 2.7–27.5°
*c* = 13.9069 (3) Å	µ = 0.24 mm^−^^1^
α = 103.719 (1)°	*T* = 173 K
β = 91.280 (1)°	Block, colourless
γ = 106.973 (1)°	0.36 × 0.20 × 0.17 mm
*V* = 711.50 (3) Å^3^	

### Data collection

**Table:**

Bruker SMART APEXII CCD diffractometer	3257 independent reflections
Radiation source: rotating anode	2971 reflections with *I* > 2σ(*I*)
graphite multilayer	*R*_int_ = 0.023
Detector resolution: 10.0 pixels mm^-1^	θ_max_ = 27.5°, θ_min_ = 1.5°
φ and ω scans	*h* = −8→8
Absorption correction: multi-scan (*SADABS*; Bruker, 2009)	*k* = −10→10
*T*_min_ = 0.919, *T*_max_ = 0.960	*l* = −16→18
12726 measured reflections	

### Refinement

**Table:**

Refinement on *F*^2^	Primary atom site location: structure-invariant direct methods
Least-squares matrix: full	Secondary atom site location: difference Fourier map
*R*[*F*^2^ > 2σ(*F*^2^)] = 0.035	Hydrogen site location: difference Fourier map
*wR*(*F*^2^) = 0.098	H-atom parameters constrained
*S* = 1.06	*w* = 1/[σ^2^(*F*_o_^2^) + (0.0509*P*)^2^ + 0.2693*P*] where *P* = (*F*_o_^2^ + 2*F*_c_^2^)/3
3257 reflections	(Δ/σ)_max_ = 0.001
193 parameters	Δρ_max_ = 0.31 e Å^−^^3^
0 restraints	Δρ_min_ = −0.29 e Å^−^^3^

### Special details

**Table:**

Geometry. All esds (except the esd in the dihedral angle between two l.s. planes) are estimated using the full covariance matrix. The cell esds are taken into account individually in the estimation of esds in distances, angles and torsion angles; correlations between esds in cell parameters are only used when they are defined by crystal symmetry. An approximate (isotropic) treatment of cell esds is used for estimating esds involving l.s. planes.
Refinement. Refinement of F^2^ against ALL reflections. The weighted R-factor wR and goodness of fit S are based on F^2^, conventional R-factors R are based on F, with F set to zero for negative F^2^. The threshold expression of F^2^ > 2sigma(F^2^) is used only for calculating R-factors(gt) etc. and is not relevant to the choice of reflections for refinement. R-factors based on F^2^ are statistically about twice as large as those based on F, and R- factors based on ALL data will be even larger.

### Fractional atomic coordinates and isotropic or equivalent isotropic displacement parameters (Å^2^)

**Table:**

	*x*	*y*	*z*	*U*_iso_*/*U*_eq_	
S1	0.41807 (5)	0.81791 (4)	0.16087 (2)	0.02634 (11)	
F1	0.65786 (15)	0.26222 (13)	0.09940 (8)	0.0430 (2)	
O1	0.81464 (14)	0.96033 (13)	0.39233 (7)	0.0267 (2)	
O2	0.21185 (17)	0.84474 (15)	0.16213 (8)	0.0370 (3)	
C1	0.5371 (2)	0.86225 (17)	0.28116 (10)	0.0233 (3)	
C2	0.4752 (2)	0.79696 (17)	0.36835 (9)	0.0225 (3)	
C3	0.2935 (2)	0.70147 (17)	0.40006 (10)	0.0249 (3)	
C4	0.3090 (2)	0.67041 (18)	0.49378 (10)	0.0272 (3)	
H4	0.1881	0.6052	0.5167	0.033*	
C5	0.4919 (2)	0.72936 (18)	0.55638 (10)	0.0275 (3)	
C6	0.6698 (2)	0.82929 (18)	0.52602 (10)	0.0276 (3)	
H6	0.7961	0.8739	0.5670	0.033*	
C7	0.6540 (2)	0.86042 (17)	0.43328 (10)	0.0240 (3)	
C8	0.7386 (2)	0.95919 (17)	0.30056 (10)	0.0250 (3)	
C9	0.0898 (2)	0.6369 (2)	0.33833 (12)	0.0338 (3)	
H9A	0.0765	0.5220	0.2909	0.051*	
H9B	0.0813	0.7251	0.3019	0.051*	
H9C	−0.0209	0.6216	0.3819	0.051*	
C10	0.4951 (2)	0.6840 (2)	0.65539 (11)	0.0337 (3)	
H10A	0.5080	0.5635	0.6458	0.051*	
H10B	0.3676	0.6880	0.6849	0.051*	
H10C	0.6119	0.7710	0.6998	0.051*	
C11	0.8879 (2)	1.0612 (2)	0.24364 (12)	0.0327 (3)	
H11A	0.8162	1.0663	0.1831	0.049*	
H11B	0.9924	1.0016	0.2255	0.049*	
H11C	0.9535	1.1832	0.2847	0.049*	
C12	0.3746 (2)	0.57927 (18)	0.12654 (9)	0.0239 (3)	
C13	0.1800 (2)	0.4675 (2)	0.08789 (11)	0.0309 (3)	
H13	0.0697	0.5155	0.0843	0.037*	
C14	0.1480 (2)	0.2839 (2)	0.05441 (12)	0.0365 (3)	
H14	0.0148	0.2062	0.0282	0.044*	
C15	0.3083 (2)	0.2136 (2)	0.05897 (11)	0.0342 (3)	
H15	0.2871	0.0883	0.0368	0.041*	
C16	0.4994 (2)	0.33028 (19)	0.09652 (10)	0.0291 (3)	
C17	0.5392 (2)	0.51277 (18)	0.13067 (10)	0.0257 (3)	
H17	0.6733	0.5897	0.1559	0.031*	

### Atomic displacement parameters (Å^2^)

**Table:**

	*U*^11^	*U*^22^	*U*^33^	*U*^12^	*U*^13^	*U*^23^
S1	0.03275 (19)	0.02625 (19)	0.02560 (18)	0.01438 (14)	0.00475 (13)	0.01027 (13)
F1	0.0476 (5)	0.0378 (5)	0.0512 (6)	0.0265 (4)	0.0033 (4)	0.0091 (4)
O1	0.0243 (5)	0.0270 (5)	0.0291 (5)	0.0065 (4)	0.0055 (4)	0.0090 (4)
O2	0.0390 (6)	0.0406 (6)	0.0410 (6)	0.0246 (5)	0.0023 (5)	0.0131 (5)
C1	0.0275 (6)	0.0211 (6)	0.0243 (6)	0.0105 (5)	0.0065 (5)	0.0072 (5)
C2	0.0275 (6)	0.0191 (6)	0.0230 (6)	0.0103 (5)	0.0058 (5)	0.0053 (5)
C3	0.0268 (6)	0.0205 (6)	0.0282 (6)	0.0084 (5)	0.0064 (5)	0.0059 (5)
C4	0.0308 (7)	0.0228 (6)	0.0296 (7)	0.0083 (5)	0.0102 (5)	0.0087 (5)
C5	0.0380 (7)	0.0230 (6)	0.0245 (6)	0.0132 (6)	0.0081 (5)	0.0065 (5)
C6	0.0309 (7)	0.0260 (7)	0.0256 (6)	0.0098 (5)	0.0022 (5)	0.0049 (5)
C7	0.0260 (6)	0.0198 (6)	0.0266 (6)	0.0076 (5)	0.0069 (5)	0.0056 (5)
C8	0.0288 (6)	0.0222 (6)	0.0275 (6)	0.0116 (5)	0.0081 (5)	0.0077 (5)
C9	0.0266 (7)	0.0377 (8)	0.0364 (8)	0.0053 (6)	0.0041 (6)	0.0140 (6)
C10	0.0457 (8)	0.0340 (8)	0.0269 (7)	0.0167 (6)	0.0083 (6)	0.0119 (6)
C11	0.0302 (7)	0.0338 (8)	0.0402 (8)	0.0117 (6)	0.0134 (6)	0.0179 (6)
C12	0.0294 (6)	0.0253 (6)	0.0199 (6)	0.0108 (5)	0.0045 (5)	0.0082 (5)
C13	0.0278 (7)	0.0373 (8)	0.0286 (7)	0.0108 (6)	0.0026 (5)	0.0096 (6)
C14	0.0326 (7)	0.0347 (8)	0.0353 (8)	0.0009 (6)	0.0028 (6)	0.0075 (6)
C15	0.0463 (9)	0.0246 (7)	0.0302 (7)	0.0073 (6)	0.0076 (6)	0.0081 (6)
C16	0.0370 (7)	0.0313 (7)	0.0257 (6)	0.0176 (6)	0.0061 (5)	0.0105 (6)
C17	0.0280 (6)	0.0276 (7)	0.0230 (6)	0.0103 (5)	0.0025 (5)	0.0072 (5)

### Geometric parameters (Å, °)

**Table:**

S1—O2	1.4914 (11)	C9—H9A	0.9800
S1—C1	1.7542 (14)	C9—H9B	0.9800
S1—C12	1.8023 (14)	C9—H9C	0.9800
F1—C16	1.3567 (16)	C10—H10A	0.9800
O1—C8	1.3648 (16)	C10—H10B	0.9800
O1—C7	1.3839 (15)	C10—H10C	0.9800
C1—C8	1.3584 (19)	C11—H11A	0.9800
C1—C2	1.4573 (17)	C11—H11B	0.9800
C2—C7	1.3947 (19)	C11—H11C	0.9800
C2—C3	1.4019 (18)	C12—C13	1.385 (2)
C3—C4	1.3918 (19)	C12—C17	1.3897 (18)
C3—C9	1.5059 (19)	C13—C14	1.392 (2)
C4—C5	1.401 (2)	C13—H13	0.9500
C4—H4	0.9500	C14—C15	1.383 (2)
C5—C6	1.388 (2)	C14—H14	0.9500
C5—C10	1.5075 (18)	C15—C16	1.375 (2)
C6—C7	1.3790 (19)	C15—H15	0.9500
C6—H6	0.9500	C16—C17	1.377 (2)
C8—C11	1.4851 (18)	C17—H17	0.9500
			
O2—S1—C1	112.14 (6)	H9A—C9—H9C	109.5
O2—S1—C12	106.48 (7)	H9B—C9—H9C	109.5
C1—S1—C12	97.57 (6)	C5—C10—H10A	109.5
C8—O1—C7	106.56 (10)	C5—C10—H10B	109.5
C8—C1—C2	107.30 (12)	H10A—C10—H10B	109.5
C8—C1—S1	118.53 (10)	C5—C10—H10C	109.5
C2—C1—S1	133.60 (10)	H10A—C10—H10C	109.5
C7—C2—C3	118.64 (12)	H10B—C10—H10C	109.5
C7—C2—C1	104.16 (11)	C8—C11—H11A	109.5
C3—C2—C1	137.13 (12)	C8—C11—H11B	109.5
C4—C3—C2	116.41 (12)	H11A—C11—H11B	109.5
C4—C3—C9	120.70 (12)	C8—C11—H11C	109.5
C2—C3—C9	122.88 (12)	H11A—C11—H11C	109.5
C3—C4—C5	124.05 (12)	H11B—C11—H11C	109.5
C3—C4—H4	118.0	C13—C12—C17	121.50 (13)
C5—C4—H4	118.0	C13—C12—S1	118.67 (10)
C6—C5—C4	119.25 (12)	C17—C12—S1	119.56 (10)
C6—C5—C10	120.47 (13)	C12—C13—C14	119.16 (13)
C4—C5—C10	120.28 (13)	C12—C13—H13	120.4
C7—C6—C5	116.58 (13)	C14—C13—H13	120.4
C7—C6—H6	121.7	C15—C14—C13	120.62 (14)
C5—C6—H6	121.7	C15—C14—H14	119.7
C6—C7—O1	124.21 (12)	C13—C14—H14	119.7
C6—C7—C2	124.97 (12)	C16—C15—C14	118.08 (14)
O1—C7—C2	110.81 (11)	C16—C15—H15	121.0
C1—C8—O1	111.15 (11)	C14—C15—H15	121.0
C1—C8—C11	133.60 (13)	F1—C16—C15	118.27 (13)
O1—C8—C11	115.25 (12)	F1—C16—C17	118.10 (13)
C3—C9—H9A	109.5	C15—C16—C17	123.62 (13)
C3—C9—H9B	109.5	C16—C17—C12	117.01 (13)
H9A—C9—H9B	109.5	C16—C17—H17	121.5
C3—C9—H9C	109.5	C12—C17—H17	121.5
			
O2—S1—C1—C8	−136.09 (11)	C1—C2—C7—C6	−179.12 (12)
C12—S1—C1—C8	112.63 (11)	C3—C2—C7—O1	−176.35 (11)
O2—S1—C1—C2	53.83 (14)	C1—C2—C7—O1	1.16 (14)
C12—S1—C1—C2	−57.46 (13)	C2—C1—C8—O1	1.05 (15)
C8—C1—C2—C7	−1.32 (14)	S1—C1—C8—O1	−171.44 (8)
S1—C1—C2—C7	169.56 (11)	C2—C1—C8—C11	−178.84 (14)
C8—C1—C2—C3	175.47 (15)	S1—C1—C8—C11	8.7 (2)
S1—C1—C2—C3	−13.7 (2)	C7—O1—C8—C1	−0.33 (14)
C7—C2—C3—C4	−2.87 (18)	C7—O1—C8—C11	179.58 (11)
C1—C2—C3—C4	−179.32 (14)	O2—S1—C12—C13	13.52 (12)
C7—C2—C3—C9	176.19 (13)	C1—S1—C12—C13	129.35 (11)
C1—C2—C3—C9	−0.3 (2)	O2—S1—C12—C17	−172.39 (10)
C2—C3—C4—C5	0.5 (2)	C1—S1—C12—C17	−56.55 (11)
C9—C3—C4—C5	−178.63 (13)	C17—C12—C13—C14	1.4 (2)
C3—C4—C5—C6	1.8 (2)	S1—C12—C13—C14	175.37 (11)
C3—C4—C5—C10	−177.97 (12)	C12—C13—C14—C15	−0.4 (2)
C4—C5—C6—C7	−1.44 (19)	C13—C14—C15—C16	−0.6 (2)
C10—C5—C6—C7	178.32 (12)	C14—C15—C16—F1	−178.79 (12)
C5—C6—C7—O1	178.58 (12)	C14—C15—C16—C17	0.7 (2)
C5—C6—C7—C2	−1.1 (2)	F1—C16—C17—C12	179.73 (11)
C8—O1—C7—C6	179.71 (12)	C15—C16—C17—C12	0.3 (2)
C8—O1—C7—C2	−0.57 (14)	C13—C12—C17—C16	−1.32 (19)
C3—C2—C7—C6	3.4 (2)	S1—C12—C17—C16	−175.24 (10)

### Hydrogen-bond geometry (Å, °)

**Table:**

*D*—H···*A*	*D*—H	H···*A*	*D*···*A*	*D*—H···*A*
C11—H11B···O2^i^	0.98	2.30	3.2727 (18)	172.
C10—H10A···Cg2^ii^	0.98	2.74	3.655 (2)	155.

Symmetry codes: (i) *x*+1, *y*, *z*; (ii) −*x*+1, −*y*+1, −*z*+1.
